# Reproduction results in parallel changes of oxidative stress and immunocompetence in a wild long-living mammal—edible dormouse *Glis glis*

**DOI:** 10.1098/rsbl.2024.0257

**Published:** 2024-10-30

**Authors:** Karolina Iwińska, Jan S. Boratyński, Aneta Książek, Joanna Błońska, Zbigniew Borowski, Marek Konarzewski

**Affiliations:** ^1^ Doctoral School of University of Białystok, Białystok, Poland; ^2^ Mammal Research Institute, Polish Academy of Sciences, Białowieża, Poland; ^3^ Faculty of Biology, University of Białystok, Białystok, Poland; ^4^ Forest Research Institute, Sękocin Stary, Poland

**Keywords:** cost of reproduction, oxidative balance, immunocompetence, longevity, trade-off

## Abstract

Oxidative stress (OS) and impaired immune function (IF) have been proposed as key physiological costs of reproduction. The relationship between OS and IF remains unresolved, particularly in long-living iteroparous species. We studied physiological markers of maintenance (OS, IF markers) in lactating, post-lactating and non-lactating females of edible dormice—a long-living rodent. We predicted the OS balance and IF to be compromised by lactation, especially in older females expected to face stronger trade-offs between life functions. We found that the age predictor (body size) correlated negatively with white blood cell level (WBC), positively with neutrophils to lymphocytes ratio and had no effect on OS markers. Oxidative damage markers (reactive oxygen metabolites (ROMs); but not antioxidant capacity) and body size-adjusted WBC were the lowest in lactating, higher in post-lactating and the highest in non-lactating females. Body size/age did not affect this correlation suggesting a similar age-independent allocation strategy during reproduction in this species. The path analysis testing the causal relationship between ROMs and WBC revealed that IF is more likely to affect OS than *vice versa*. Our study indicates the trade-off between crucial life functions during reproduction and suggests that immunosuppression reduces the risk of OS; therefore, mitigating oxidative costs of reproduction.

## 1. Introduction

The physiological burden of reproduction results in an elevated metabolism, which may lead to oxidative stress (OS) [1–3] and/or impaired immune function (IF) [4]. Both OS and reduced IF have been proposed as physiological manifestations of reproductive costs, ultimately affecting the evolution of life-history strategies [1,5,6]. Studies on the effect of reproduction on OS gave inconsistent results, with breeding animals manifesting reduced rather than elevated OS, which can be explained by oxidative shielding—a preemptively activated mechanism reducing oxidative damage to protect mother and offspring [3]. Mixed results were also found for the effect of reproduction on IF with detected immunosuppression [4] or a lack of apparent trade-off [6,7]. Furthermore, despite being theoretically linked, only a few studies simultaneously analyse OS and IF and their association in the context of reproduction [8–11].

Unequivocal results of studies on the physiological manifestations of the costs of reproduction can result from a complex interplay between OS and IF. When oxidative balance is sustained, reactive oxygen species (ROS) as signalling agents regulate (both trigger or suppress) inflammatory mechanisms and the immune responses [12,13]. Maintenance of competent immune defences involves ROS-associated costs resulting from the proliferation of leukocytes and the production of molecules, which participate in immune response, as well as the deliberate production of ROS by phagocytes to kill pathogens [14]. Therefore, immune response and maintenance of competent immune status may upset the oxidative balance, resulting in OS [15], while simultaneously OS leads to impaired IF [16,17].

Age-related deterioration of physiological functions affects multiple aspects of self-maintenance and reproduction. Accumulation of oxidative damage to macromolecules is responsible for immunoaging [18] and a decline in fertility [19]. The terminal investment hypothesis predicts that individuals should increase reproductive efforts later in life as prospects for future survival decrease [20]. Therefore, trade-offs between crucial life functions during breeding should be more apparent in older individuals within a population and should be expected in long-living species [20]. Most studies on the costs of reproduction relied on short-living organisms [1,21], which simply may not invest in effective but costly mechanisms preventing oxidative damage (but see [22]). Conversely, it is the long-living species, especially free-ranging ones, that need to balance available resources, should prioritize self-maintenance for survival and must remedy current reproductive costs [14,23]. Long-living wild animals are characterized by slow life histories as high survival and a lower rate of reproduction adjusted to fluctuating resources availability [24]. This is also associated with lower ROS production and higher antioxidant capacity (OXY) when compared with fast life-history strategies [25] (but see [26]). Long-living organisms are also expected to maintain more efficient immune defences than short-living ones [5,14], even during breeding when the mitigation of oxidative costs of reproduction is required.

We studied OS and IF as manifestations of the costs of reproduction in free-ranging female edible dormice *Glis glis* that can live up to 14 years under natural conditions [27]. Dormice are hibernators with very short activity season (2–4 months) [28], which must suffice to restore depleted reserves after the winter season, reproduce and prepare for the next several months without feeding opportunities. Moreover, hibernation-related metabolic adjustments are associated with OS [29] and severely decreased immunocompetence [30]. We hypothesize that because of limited resources, wild female dormice are not able to simultaneously invest in reproduction, preventing oxidative damage and sustaining a competent immune system. We expected reduced oxidative damage and high OXY, with simultaneously decreased leukocyte count and increased inflammation in breeding (lactating and post-lactating) when compared with non-lactating dormice. We also expected that because of the progressive decline in somatic performance in older animals, oxidative damage will increase, while IF will be reduced with age. Thus, we expected interaction between the dormice age and the reproductive status, and a steeper relationship between IF and OS in older females. To test this, we measured the total white blood cell (WBC) level and used the neutrophils to lymphocytes ratio (N/L) as markers of infection and inflammation status and reactive oxygen metabolites (ROMs) and OXY as markers of OS. We predicted that all the above effects will be more apparent in lactating than non-lactating females, with intermediate values in post-lactating females since they may already allocate energy into repair mechanisms. Since ROS are immunosignalling agents [13], which suppress or trigger IF, we predicted that if there is a causal link between OS and IF, the variation in OS markers will better explain the variation in IF than the other way around.

## 2. Materials and methods

### (a) Animals

The edible dormice were captured in the Kozienice Forest (51°30′ N, 21°27′ E), central Poland (details in [31,32]). Between August and September of each year 2019–2021, females (in total: 21 lactating, 17 post-lactating and 44 non-lactating) were transported to the laboratory of Forest Research Institute in Sękocin Stary. Lactating females were at the end of the lactation; their around one-month-old juveniles had fur and moved freely; however, were still fed with mother’s milk. Post-lactating females were identified by baldness around sclerotized nipples, while the non-lactating dormice were identified as those with fur-covered non-sclerotized nipples—indicating no lactation in the given season. The animals were kept in rodent cages (1290D, Tecniplast, Buguggiate, Italy) under natural photoperiod and ambient temperature approximately 20°C with water and food provided *ad libitum* (Megan, Kraków, Poland and apples). Post-lactating and non-lactating females were kept individually. Lactating females were kept together with their juveniles and separated only for a period of blood sampling and convalescence (maximum 2 h). During this time, juveniles were supplemented with milk powder (The District Dairy Cooperative, Siedlce, Poland). After approximately 4 days in the laboratory, all animals were released at the place of capture. All animals were marked individually (RF-IDW-1, CBDZOE, Poland). No single female was measured twice during a 3 year study.

### (b) Age determination

**Figure 1.**
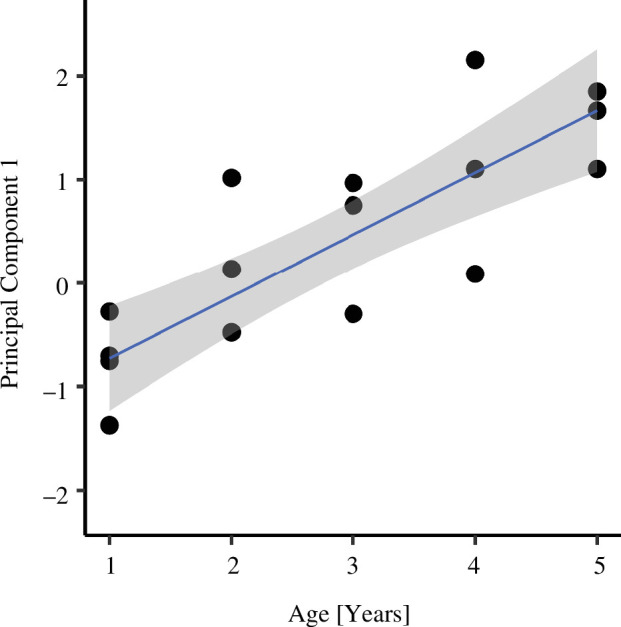
Correlation between principal component 1 (explaining 78% of the variation in body mass and size, estimated as head width) and age of 17 female edible dormice marked as juveniles during previous studies.

In edible dormouse, most of the known age determination methods are subjective and thus unreliable [33,34]. The indisputable age division is to distinguish juveniles (before the first hibernation) from adults. Since we captured and marked animals during previous studies [31,32] when they were juveniles, we knew the exact age of 17 out of 82 studied females. In all the studied animals, we measured head width in the broadest point—zygomatic arches with a calliper to the nearest 0.1 mm (Measy DG, Ecotone) and body mass with a resolution of 2 g (Pesola 40 300 Medio Line, Pesola^®^, Switzerland). In small mammals, the body size reflected by body mass and individuals’ linear dimensions is a common age determinant [35]. Since the common variance of head width and body mass in the studied population correlated strongly with the age of female dormice marked previously as juveniles [32] (figure 1), we consider it as an age predictor for all the studied animals.

### (c) Blood sampling

Blood sampling protocol was performed after approximately 30 h spent in the animal room to reduce stress associated with capture and transport. Edible dormice are nocturnal rodents and thus we took blood samples during the circadian activity phase (starting an hour after sunset) since some of non-lactating individuals used torpor during a day [36], which can influence immunological parameters [37]. During the procedure (lasting < 3 min), animals were anaesthetized under a 2% isoflurane (Iso-Vet) and atmospheric air. Blood samples (approx. 50 µl) were taken from the retro-orbital sinus using heparinized microhaematocrit capillary tubes (VITREX Medical A/S, Denmark). To establish the leukocyte status, a blood smear was prepared from 4 µl of full blood. In 15 min after sampling, blood was centrifuged at 13 000 rpm for 20 min at 4°C, and plasma was immediately stored at −80°C until the assays were performed.

### (d) The oxidative status

The dROM Assay Kit (Diacron, Grosseto, Italy) was used to measure the level of ROMs in the blood plasma—result of exposure of organic macromolecules to ROS (especially hydroperoxides that maintain oxidizing properties and are one of the major immunosignalling agents) [13]. ROMs are a reliable indicator of oxidative status in organisms [38]. The assay was performed according to the manufacturer’s instructions, with minor modifications (as in [39]). Briefly, 4 µl plasma samples were diluted with 200 μl of a solution of chromogen and acetate buffer (pH 4.8), with which derivatives of ROMs form a coloured compound. After 90 min of incubation at 37°C with gentle shaking, samples were shortly centrifuged to precipitate any formed sediment and transferred to a 96-well plate. The absorbance was read (Multiskan FC Microplate Photometer, Thermo Fisher Scientific, USA) at 493 nm. The absorbance is directly proportional to the concentration of ROMs, expressed as ‘Carratelli units’ (CARR U), where 1 CARR U is equivalent to 0.08 mg of H_2_O_2_ 100 ml^−1^.

The OXY was measured by the OXY-Adsorbent Test (Diacron, Grosseto, Italy), which evaluates endogenous and exogenous chemically active scavengers circulating in the plasma. This test measures the ability of antioxidants to cope with the oxidant action of hypochlorous acid (HClO). We performed the analysis (as in [39]) with the use of 2 μl plasma diluted with distilled water in a 1 : 100 ratio. Then 200 μl of HClO solution was added. After incubation (37°C, 10 min) and adding 2 μl of chromogen, the absorbance was read at 493 nm. OXY values are given in mmol HClO ml^−1^ of plasma.

All measurements were conducted in duplicate. Intra-plate variation was 4.63% and 7.03% for ROMs and OXY, respectively. Inter-plate variation based on a standard sample repeated over plates was 8.98% and 14.06% for ROMs and OXY, respectively.

### (e) Leukocyte counts

The WBC level, estimated numbers of neutrophils and lymphocytes for known amount of erythrocytes in the bloodstream, and N/L ratio were chosen as markers of IF. Those parameters are a measure of infection and inflammation status in animals [40,41]. The blood smears were prepared by the two-slide wedge technique. They were air-dried and stained according to the Pappenheim method (Giemsa stain and May–Grünwald stain, Kolchem, Poland). The WBC count in each individual was performed in 10 counts at different smear locations, which gave us a total of WBC per 100 000 RBC. In statistical analysis, we used an average of WBC numbers from 10 counts. The counting of RBC was performed using ‘CountThings’ software (v. G3.75.1, Dynamic Ventures International, Brasov).

The 100 WBC in four counts were examined on each slide under a microscope with 1000× magnification and differentiated into lymphocytes, neutrophils, eosinophils, monocytes and basophils. For each individual, neutrophils and lymphocytes were calculated as the proportion of neutrophils to lymphocytes counts per 400 leukocytes multiplied by the averaged level of WBC (mean of 10 counts of WBC level per 10 000 RBC).

All the smears were examined by the same observer (J.B.).

### (f) Statistics

All statistical analyses were performed in R program (v. 4.3.2). The data and code are available from the Dryad Digital Repository [42].

Body size was calculated as a common variance of head width and body mass (principal component 1 (PC1)) obtained in principal component analysis (PCA). The relation between body size and age was tested using Pearson correlation.

Since dormice were sampled from the population during three subsequent years, initially we used mixed effect models (LME) with a restricted maximum likelihood for OXY, ROMs, WBC, neutrophils, lymphocytes and N/L using the function ‘lmer’ of R-package ‘lme4’. Since year was not a significant predictor for most of dependent variables, we then also calculated linear models (LM) assuming random sampling. In LMEs and LMs, reproductive status (lactation, post-lactation and non-lactation) was included as a factor and body size as a covariate. In LMEs year was considered as a random effect. We used the function ‘lrtest’ of R-package ‘lmtest’ to test if year significantly explain variance. We also tested the interaction between reproductive status and body size in each model. WBC, neutrophils, lymphocytes and N/L were log_10_-transformed before analysis to reduce the heteroscedasticity of model residuals. The analysis of variance (ANOVA) with type II or type III sum of squares was used to test the influence of reproductive status and body size on each response variable based on models without or with interaction included, respectively. Tukey HSD test was used for *post-hoc* comparisons between reproductive categories. All continuous variables were centred and scaled prior to analysis (subtracted from the population mean and divided by the population standard deviation). Since we had clear predictions for between groups differences (gradual differences between reproductive status) we did not adjust alpha for the falsepositive detection rate to avoid type I error in statistical reasoning [43]. Estimated marginal means with 95% confidence intervals were obtained using the function ‘emmeans’ of R-package ‘emmeans’.

Path model (PM) analysis was used to make an inference about the plausible causal relationship between WBC and ROMs using function ‘psem’ of R-package ‘piecewiseSEM’ [44] to conclude whether changes in one function have consequences for the other. Two PMs were compared, one with variation in ROMs assumed as a predictor for variation in WBC and the other, with variance in WBC predicting variance of ROMs. Each PM included two LMEs, where ROMs or WBC (log_10_-transformed) were dependent variables, year was a random effect and body size was a covariate for WBC. Corrected Akaike information criterion (AICc) was used to compare the two PM models.

## 3. Results

### (a) Body size–age predictor

PC1 explained 78% of the variation in body mass and head width (electronic supplementary material, table S1). PC1 correlated positively with known age of a subset of 17 dormice that were individually marked during previous studies [31,32] as juveniles (*r* = 0.83, *n* = 17, *p* < 0.001; figure 1). Therefore, in subsequent analyses, we used PC1 as a proxy of age (henceforth: PC1-body size/age) of all individuals.

### (b) Oxidative stress and immunocompetence

**Table 1.** Analysis of variance based on mixed effect model conducted for physiological markers of oxidative stress and immune status in lactating, post-lactating and non-lactating edible dormouse females of different body size/age (PC1-common variance of head width and body mass obtained in principal component analysis).

category	dependent factor	reproductive status	PC1 - body size/age	reproductive status × PC1 body size/age
oxidative status	antioxidant capacity	*F*_2,77_ = 0.08, *p* = 0.921	F_1,77_ = 0.28, *p* = 0.600	F_2,74_ = 0.98, *p* = 0.381
	oxidative damage	***F*_2,65_ = 25.34, *p* < 0.001**	*F*_1,78_ = 1.69, *p* = 0.198	F_2,76_ = 1.07, *p* = 0.350
immunology	white blood cells level	***F*_2,76_ = 6.53, *p* = 0.002**	***F*_1,78_ = 12.47, *p* < 0.001**	F_2,75_= 0.30, *p* = 0.739
	neutrophil to lymphocyte ratio	*F*_2,77_ = 0.63, *p* = 0.536	***F*_1,77_ = 6.13, *p* = 0.016**	F_2,75_ = 0.44, *p* = 0.646
	estimated neutrophil numbers	*F*_2_,_77_ = 0.77, *p* = 0.466	*F*_1,77_ = 0.04, *p* = 0.843	F_2,75_ = 0.75, *p* = 0.474
	estimated lymphocyte numbers	***F*_2,71_ = 7.89, *p* < 0.001**	***F*_1,78_ = 16.28, *p* < 0.001**	F_2,75_ = 0.21, *p* = 0.814

Bold indicates significance.

**Figure 2.**
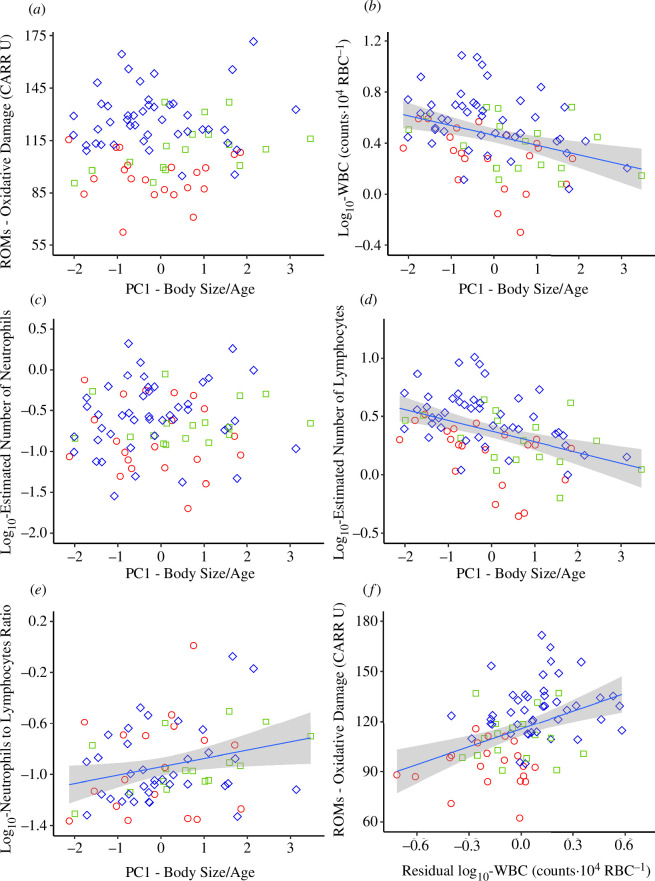
Relationship between (*a*) oxidative damage (ROMs), (*b*) white blood cells (WBC) level, (*c*) estimated numbers of neutrophils, (*d*) estimated numbers of lymphocytes, (*e*) neutrophils to lymphocytes ratio with body size/age (PC1 - common variance of head width and body mass obtained in principal component analysis) and (*f*) residual WBC (not explained by PC1-body size/age) and ROMs, shown for lactating (red circle), post-lactating (green square) and non-lactating (blue diamond) edible dormouse females.

The ROMs significantly differed between females of different reproductive status (table 1), being the lowest in lactating, higher in post-lactating and the highest in non-lactating females (figure 2*a*; table 2, electronic supplementary material, table S2). In contrast, OXY was not affected by reproductive status (tables 1 and 2). The PC1-body size/age did not affect either ROMs (figure 2a) or OXY (table 1).

**Table 2.** Estimated marginal means and 95% confidence intervals for physiological markers of oxidative stress and immune status in different reproductive status groups of female edible dormice. Marginal means labelled with different letters differ significantly at *p* ≤ 0.05 (Tukey HSD test). Summary statistics were obtained on mixed or linear models, respectively. Back-transformed values are given for log_10_ transformed data.

dependent factor	model	lactating	post-lactating	non-lactating
antioxidant capacity (mmol HClO ml^−1^)	mixed linear	321.0(223.0-419.0)^A^ 297.0(272.0-323.0)^A^	323.0(226.0-419.0)^A^ 322.0(293.0-351.0)^A^	317.0(208.0-426.0)^A^ 320.0(303.0-338.0)^A^
oxidative damage (CARR U)	mixed linear	94.9(84.7-105.0)^A^ 94.2(87.5-101.0)^A^	110.1(100.8-119.0)^B^ 109.9(102.3-118.0)^B^	128.0(118.4-138.0)^C^ 127.8(123.2-132.0)^C^
body size-adjusted white blood cell level (counts ·10^4^ RBC^−1^)	mixed linear	2.07(1.37-3.13)^A^ 1.87(1.51-2.32)^A^	2.64(1.79-3.91)^A^ 2.70(2.11-3.44)^B^	3.50(2.22-5.52)^B^ 3.66(3.16-4.26)^C^
neutrophil to lymphocyte ratio	mixed linear	1.29(0.09-17.78)^A^ 0.10(0.07-0.14)^A^	0.94(0.07-12.30)^A^ 0.10(0.06-0.15)^A^	0.67(0.03-13.49)^A^ 0.09(0.07-0.11)^A^
estimated neutrophil numbers	mixed linear	0.54(0.04–8.07)^A^ 0.14(0.10–0.22)^A^	0.80(0.06–11.14)^A^ 0.22(0.14–0.34)^A^	1.15(0.05–25.53)^A^ 0.27(0.20–0.36)^A^
estimated lymphocyte numbers	mixed linear	0.24(0.07–0.90)^A^ 1.49(1.19–1.85)^A^	0.76(0.23–2.54)^A^ 2.21(1.72–2.84)^A^	2.23(0.60–8.35)^A^ 3.03(2.60–3.52)^A^

The WBC level decreased with PC1-body size/age (*β* ± s.e. = −0.34 ± 0.09; figure 2*b*). Neutrophils did not change, while lymphocytes decreased with PC1-body size/age (*β* ± s.e. = −0.38 ± 0.09); therefore, inflammatory marker— N/L ratio increased with size thus the age of females (*β* ± s.e. = 0.25 ± 0.10; figure 2*c*–*e*, table 1). The N/L ratio did not differ between lactating, post-lactating and non-lactating females (tables 1 and 2). The PC1-body size/age-adjusted WBC counts differed significantly with respect to reproductive status, being the highest in non-lactating, intermediate in post-lactating and the lowest in lactating females (figure 2*b*, table 2, electronic supplementary material table S2).

The PC1-body size/age and reproductive status interaction was not significant for all studied markers (table 1).

The PM assuming that variation in WBC is a predictor of variation in ROMs explained variance better (AICc = 715, *k* = 4, *n* = 82) than the model assuming the oposite (AICc = 737, *k* = 8, *n* = 82) at a statistically significant level (models ΔAICc > 2). According to this more informative PM, variation in WBC (not explained by its negative correlation with PC1—body size/age; *r* = −0.35, *p* < 0.001) correlated positively with ROMs (*r* = 0.30, *p* = 0.009; figure 2*d*).

## 4. Discussion

The results of this study indicate the existence of a trade-off between maintenance of oxidative balance and sustaining immunocompetence during reproduction in a long-lived mammalian species. Age did not affect the positive correlation between OS and IF resulting from variation in breeding status. This suggests that the females studied used similar allocation strategies irrespective of their age. Moreover our statistical modelling indicates that the changes in IF during reproduction shapes OS rather than *vice versa*.

Reproduction is associated with oxidative shielding—a protective mechanism quenching OS, mainly understood as increased antioxidant protection [3,45]. Surprisingly, although lactating dormice had lower ROMs than non-lactating ones, their OXY was not affected by breeding status. A similar pattern was noted in a few other studies on long-living species [45,46]. This implies that other yet unidentified mechanisms besides antioxidant protection are responsible for quenching OS during reproduction.

The higher WBC level in non-lactating than in lactating and post-lactating females could be a manifestation of inflammation or infection preventing them from reproduction. However, this should be accompanied by a simultaneous increase in neutrophil level [47], resulting in a significantly increased N/L ratio, which we did not observe (tables 1 and 2). Alternatively, the low WBC counts in breeding females (lactating and post-lactating) may indicate immunosuppression during reproduction [4,5], which agrees with the lack of the effect of breeding status on the N/L ratio.

The correlated reduction of ROMs and WBC levels during lactation suggests the adaptive suppression of immune defense likely allowing for the balancing of OS during breeding. Only one study, conducted on reproducing wild little auk *Alle alle*, aimed to directly link immunity and OS during reproduction, supporting our results [11]. In these long-living birds, the ROMs were also positively correlated with WBC level. Our statistical causal modelling suggests that variance in WBC explains the variance in ROMs. Therefore, a decreased IF during reproduction is likely a mechanism to reduce excessive ROS production. Such an idea has already been proposed in fish models [48] and can be supported by veterinary studies that indicated reduced phagocytic oxidative burst (production of ROS to kill pathogens) during pregnancy and birth period in cattle [49,50].

Age affects adaptive and innate immune responses by reducing the relative abundance of WBC and negatively affecting the functions of those cells [51,52]. In accordance with the literature [53], in the studied dormice, leukocyte number declined, while the N/L ratio increased with the age predictor. The N/L ratio reflects the dynamic relationship between innate and adaptive cellular immune response [39]. Therefore, we attribute its increase with body size/age to immunosenescence—the age-associated dysregulation of the IF, primarily manifested by progressive impairment of adaptive (memory-based) immune responses [52]. In agreement, we observed the decline in lymphocyte number constituting an adaptive response, with a simultaneous lack of change in the number of neutrophils, which resulted in a high N/L ratio (figure 2*c–e*), most likely associated with chronic low-grade inflammation frequently observed in senescence [52].

Despite observed age-related differences in IF, there was a lack of significant interactions between reproductive status and age predictor for tested self-maintenance markers. As the maximum age of dormice in our study was half of the maximum observed in this species (electronic supplementary material figure S1) [27], one may suggest insufficient representation of very old individuals that are indispensable to demonstrate OS-related deterioration of somatic functions [54]. However, we did notice an body size/age-related decline in IF markers, which is consistent with the literature [51–53]; therefore, we believe we noted the aging process and if there was an interaction with reproduction, we should have noted it. From this perspective, our study does not support the terminal investment hypothesis.

This study is the first to show that under natural conditions, a long-living mammal reduces oxidative damage incurred by reproduction through immunosuppression, supporting the oxidative shielding hypothesis and suggesting a universal mechanism for reducing ROS production in breeders. Our study suggests an alternative to a widely accepted antioxidant-reliant pathway—the suppression of ROS increasing functions. We suggest that since increasing OXY during reproduction is too costly under natural conditions in free-ranging animals, individuals must sacrifice other crucial self-maintenance functions, like IF, to maintain oxidative balance. Thus, here we show how important it is to consider more than a single physiological function in studies concerning the costs of reproduction, as implied by Monaghan and colleagues’ theoretical model ([2] see fig. 2 therein). Simultaneous studies on IF and OS are needed to draw correct conclusions on the actual costs of reproduction and their consequences for fitness.

## Data Availability

The data and code are available from the Dryad Digital Repository [42]. Supplementary material is available online [55].
